# Vaccine-elicited and naturally elicited antibodies differ in their recognition of the HIV-1 fusion peptide

**DOI:** 10.3389/fimmu.2024.1484029

**Published:** 2024-11-14

**Authors:** Mateo Reveiz, Kai Xu, Myungjin Lee, Shuishu Wang, Adam S. Olia, Darcy R. Harris, Kevin Liu, Tracy Liu, Andrew J. Schaub, Tyler Stephens, Yiran Wang, Baoshan Zhang, Rick Huang, Yaroslav Tsybovsky, Peter D. Kwong, Reda Rawi

**Affiliations:** ^1^ Vaccine Research Center, National Institute of Allergy and Infectious Diseases, National Institutes of Health, Bethesda, MD, United States; ^2^ Computational and Systems Biology Program, Massachusetts Institute of Technology, Cambridge, MA, United States; ^3^ Department of Veterinary Biosciences, The Ohio State University, Columbus, OH, United States; ^4^ Vaccine Research Center, Electron Microscopy Unit, Cancer Research Technology Program, Frederick National Laboratory for Cancer Research, Frederick, MD, United States; ^5^ Laboratory of Cell Biology, National Cancer Institute, National Institutes of Health, Bethesda, MD, United States; ^6^ Aaron Diamond AIDS Research Center, Columbia University Vagelos College of Physicians and Surgeons, New York, NY, United States

**Keywords:** fusion peptide, HIV-1 vaccine, naturally elicited, neutralizing antibody, vaccine elicited

## Abstract

Broadly neutralizing antibodies have been proposed as templates for HIV-1 vaccine design, but it has been unclear how similar vaccine-elicited antibodies are to their naturally elicited templates. To provide insight, here we compare the recognition of naturally elicited and vaccine-elicited antibodies targeting the HIV-1 fusion peptide, which comprises envelope (Env) residues 512–526, with the most common sequence being AVGIGAVFLGFLGAA. Naturally elicited antibodies bound peptides with substitutions to negatively charged amino acids at residue positions 517–520 substantially better than the most common sequence, despite these substitutions rarely appearing in HIV-1; by contrast, vaccine-elicited antibodies were less tolerant of sequence variation, with no substitution of residues 512–516 showing increased binding. Molecular dynamics analysis and cryo-EM structural analysis of the naturally elicited ACS202 antibody in complex with the HIV-1 Env trimer with an alanine 517 to glutamine substitution suggested enhanced binding to result from electrostatic interactions with positively charged antibody residues. Overall, vaccine-elicited antibodies appeared to be more fully optimized to bind the most common fusion peptide sequence, perhaps reflecting the immunization with fusion peptide of the vaccine-elicited antibodies.

## Introduction

Two general strategies—lineage-based and epitope-based—have been proposed by which to use broadly neutralizing antibodies as templates for HIV vaccine development (1–3). In lineage-based strategies, antibodies of the same reproducible class are elicited by priming with immunogens that preferentially bind to initial recombinants, followed by boosts to ensure appropriate lineage maturation, with a final polishing step to expand the expression of the vaccine-elicited broadly neutralizing antibodies (4–7). This approach is achieving substantial success in knock-in mouse models (8, 9) and in the initial stage of priming in human clinical trials (10). In epitope-based strategies, sites of vulnerability on the HIV-1 envelope (Env) trimer are defined by antibodies with broad and potent neutralization, and immunogens focus the immune response to specific sites. Success has been achieved through diverse approaches involving (i) priming with Env trimers with *N*-linked glycans proximal to the target site removed to enhance local immunogenicity (11, 12) or (ii) priming with peptides or nanoparticle-based scaffolds that antigenically mimic the target site (13–16).

Success with epitope-based design has been particularly noteworthy at the fusion peptide (FP) site of vulnerability, with broadly neutralizing responses achieved in mice, guinea pigs, and non-human primates (17–19). This breakthrough has allowed analysis of the degree of similarity between the vaccine-elicited antibodies and the naturally elicited ones.

In this study, we measured the impact of single-point mutations on the FP, residues 512–526, using the most common FP sequence (_512_AVGIGAVFLGFLGAA_526_; FP15) on the binding of eight vaccine-elicited antibodies and three naturally elicited antibodies. The analysis was based on a PEPperMAP^®^ epitope substitution scan of all amino acid positions with the 20 canonical amino acids (20). The naturally elicited antibodies included three human antibodies: PGT151 (isolated from an elite neutralizer infected with a clade C virus; 60% breadth on 208 panel) (21), N123-VRC34.01 (isolated from a donor infected with a clade B virus; 50% breadth) (22), and ACS202 (isolated from an individual infected with a subtype B virus; 26% breadth) (23). For vaccine-elicited antibodies, we selected three murine antibodies (15), vFP1.01, vFP5.01 (elicited through vaccination with the BG505 SOSIP prime and three FP8-KLH boosts; 8.1% and 1% breadths, respectively), and vFP16.02 (BG505 SOSIP prime followed by three FP10-KLH; 31% breadth), as well as five macaque antibodies (18), 0PV-a.01, 0PV-b.01, 0PV-c.01, DF1W-a.01 (five FP10–1M6T-KLH primes followed by three Env trimer boosts; 17%, 13%, 23%, and 25% breadths), and DFPH-a.01 (two FP10-1M6T-KLH primes followed by three Env trimer boosts; 59% breadth). Select substitutions in FP were assessed for altered affinity in the context of prefusion-closed Env trimers. The cryo-EM structure of naturally elicited antibody ACS202 was determined in complex with one of the substitutions with most enhanced affinity, and molecular dynamics analysis was used to provide mechanistic insight into affinity improvement. Overall, naturally elicited antibodies showed substantially higher affinity to FP variants, most of which were not commonly present in HIV-1, whereas the vaccine-elicited antibodies appeared to be optimized to recognize the most common sequence variant of FP.

## Materials and methods

### Preparation of antibodies

In this study, a total of three human, three murine, and five macaque antibodies were generated. The DNA encoding the variable domains of the heavy and light chains for each antibody was codon optimized, synthesized, and cloned into specific VRC8400-based mammalian expression vectors. Antibodies were generated utilizing constant CH1 and CL domains that matched the species from which they were derived. The Fc domains for these antibodies were from human origin so that anti-human Fc could be used for the chip readout or sensor capture of antibodies. Plasmids carrying the individual antibody heavy- and light-chain sequences were co-transfected into Expi293 cells using Turbo293 transfection reagent following the manufacturer’s protocol. The transfected cells were then incubated in shaker incubators at 120 rpm, 37°C, and 9% CO_2_. On the second day, AbBooster medium was added to the cell cultures at 1/10th of the culture volume, and the incubation temperature was lowered to 33°C while maintaining the other conditions. The cell cultures were further incubated for an additional 5 days. After 6 days post-transfection, the cell culture supernatants were harvested. Antibodies were purified from supernatants using protein A chromatography. The purification process involved a PBS wash followed by elution with a low-pH glycine buffer. The eluted antibodies were immediately neutralized using 10% volume of 1 M Tris buffer at pH 8.0.

### PEPperMAP^®^ epitope substitution scan

The substitution scan analysis was performed as described in PEPperPRINT methods (20). Pre-staining of an AVGIGAVFLGFLGAA peptide microarray copy was done with the secondary goat anti-human IgG (Fc) DyLight 680 antibody and the control antibody in incubation buffer to investigate background interactions with the variants of wild-type (WT) peptide AVGIGAVFLGFLGAA that could interfere with the main assays. Subsequent incubation of further AVGIGAVFLGFLGAA peptide microarray copies with the synthetic human antibodies at concentrations of 1 µg/ml, 10 µg/ml, and 100 µg/ml in incubation buffer was followed by staining with secondary and control antibodies as well as by microarray readout at scanning intensities of 5/7 or 7/7 (red/green). The additional HA control peptides framing the peptide microarrays were simultaneously stained as internal quality control to confirm the assay quality and the peptide microarray integrity.

Quantification of spot intensities and peptide annotation were based on the 16-bit gray-scale tiff files at a scanning intensity of 5/7 or 7/7 that exhibit a higher dynamic range than the 24-bit colorized tiff files included in this report; microarray image analysis was done with PepSlide^®^ Analyzer. A software algorithm breaks down fluorescence intensities of each spot into raw, foreground and background signals (see “Raw Data” tabs) and calculates averaged median foreground intensities and spot-to-spot deviations of spot triplicates (see “Data Summary” tabs). Based on averaged median foreground intensities, intensity maps were generated and interactions in the peptide maps highlighted by an intensity color code with red for high and white for low spot intensities.

Correlations with differential selection were performed by extracting the relevant metrics from the Dingens and Doud manuscripts and corresponding GitHub repositories https://github.com/jbloomlab/MAP_NHP_FP_Abs/tree/master (24, 25).

### HIV-1 Env trimer production

Soluble HIV-1 Env trimers were expressed and purified as previously described (26). Briefly, plasmids encoding either BG505-RnS or its mutants, with an N-terminal single-chain Fc tag and an HRV 3C cleavage site, were transfected into HEK293F Freestyle cells. The protein was expressed for 6 days at 37°C, after which the supernatant was cleared by centrifugation and filtration. The HIV-1 trimers were purified by affinity chromatography with Protein A resin, and the Env trimer liberated from the tag by cleavage with HRV 3C. The eluted Env trimer was further purified by gel filtration on a Superdex 200 column, equilibrated in PBS. The proteins were concentrated to 1 mg/ml, flash frozen in 10% glycerol, and stored at −80°C until use.

### FP antibody binding assessment

To quantify the binding between BG505 or its mutants and FP-directed antibodies, the interaction was assessed by BLI using a ForteBio Octet HTX. IgG antibodies were loaded onto Anti-human Fc (AHC) tips at 20 µg/ml for 3 min, followed by a second baseline phase in PBS for 1 min. The tips were then dipped into wells with the HIV-1 trimers at 50 µg/ml for 5 min, followed by a dissociation phase in PBS for 5 min. Binding curves were analyzed, the background subtracted using the ForteBio Data Analysis 12.0 software, and corrected response values are reported.

### ACS202 Fab and BG505_RnS_7mut_A517E trimer preparation

Antibody ACS202 IgG was purified as described above. Briefly, the codon-optimized variable domains of heavy chains and light chains were cloned into a VRC8400 vector as previously described (27), with an HRV 3C cleavage site on the heavy-chain hinge region. The antibody was expressed by transient transfection in Expi293 cells with plasmids encoding heavy- and light-chain genes. The antibody IgG was purified from a Protein A column and then cleaved by HRV 3C protease to obtain the antibody Fab, which was purified by passing through a Protein A column and then a Superdex 200 column with PBS buffer. The BG505_RnS_7mut_A517E trimer for cryo-EM sample preparation was produced as described above.

### Cryo-EM structural analysis

The ACS202 Fab was mixed with BG505_RnS_7mut_A517E trimer at an ~4.5:1 molar ratio (Fab to trimer) at a total protein concentration of ~5 mg/ml. DDM (stock concentration of 1 mM) was added to a final concentration of 0.1 mM. 2.7 μl of the mixture was pipetted to a Quantifoil R 2/2 gold grid and vitrified with Vitrobot Mark IV (Thermo Fisher Scientific) using parameters set to 4°C, 95% humidity, no wait time, 2-s blot time, and blot force of −5. Data were acquired with a 300-kV Titan Krios (Thermo Fisher Scientific) equipped with Gatan BioQuantum K3 and operated in the super-resolution mode (0.415 Å/pixel raw unbinned, 0.83 Å/physical pixel) using serialEM (28). Data were processed using cryoSPARC 3.3 (29) for patch motion correction, patch CTF estimation, particle picking with a Blob picker, 2D classifications, *ab initio* 3D reconstructions, and homogenous and non-uniform refinements. The final reconstruction map was calculated with non-uniform refinement with C3 symmetry.

Initial rigid body fits of the HIV-1 trimer and Fab to the cryo-EM reconstructed maps were performed with Chimera (30) using the BG505 Env trimer structure from PDB: 6vi0 (31) and the ACS202 Fab structure from PDB: 6nc2 (32) as starting models. The mutations in the BG505 Env sequence were manually built with Coot (33), and the coordinates were refined to fit the electron density map by an iterative process of manual fitting with Coot (33) and real space refinement with Phenix (34). MolProbity (35) was used to evaluate the structural quality. Figures were generated with Chimera and PyMOL (36).

### Molecular dynamics simulations

Molecular dynamics (MD) simulations were performed for energetic analysis of antibody–FP complexes and antibody–Env trimer complexes. A total of 10 antibody–FP structures (0PVc.01 WT (PDB:6MQC), 0PVc.01 V513E, VRC34(PDB:5I8E), VRC34 G516M, VRC34 G514S, vFP16.02(PDB:6CDO), vFP16.02 G514S, PGT151(PDB:5FUU), PGT151 V513E, PGT151 A512Y) were analyzed, with FP sequence mutations generated by PyMOL (36) with the lowest clashing rotamer. Specifically, the PGT151–FP complex was truncated from the trimer (PDB:5FUU) and the constant region of Fab was modeled by the VRC34–FP complex structure. Cryo-EM structures of ACS202 in complex with the BG505 Env trimer and with mutant A517E were prepared for simulations by modeling missing residues using YASARA (http://www.yasara.org/) and man-5 glycosylation by CHARMM-GUI Glycan Reader and Modeler (37). CHARMM-GUI (38, 39) was used to generate necessary setups for the MD simulations for the 10 antibody–FP complexes and two BG505_ACS202 mutants. Initial structures were solvated in a 100 Å × 100 Å × 100 Å padded water box for the antibody–FP complexes and 197 Å × 197 Å × 197 Å for BG505_ACS202 mutants. Both systems were neutralized by adding 150 mM of KCl. The NAMD2.13 engine (40), with the CHARMM36 force field (41, 42) used to run MD simulations. The water molecules were represented as TIP3P water parameterization (43). The electrostatic interactions under periodic boundary conditions were computed using particle-mesh Ewald (PME) summation (44) with a grid spacing smaller than 1 Å. The system was performed energetic minimization by 10,000 conjugate gradient steps and equilibrated using a linear temperature gradient. The temperature of the system went up to 310 K in 125,000 steps. The length of hydrogen bonds was constrained with the SHAKE algorithm (45) with a 2-fs time step. The production step of MD was conducted for 200 ns with NPT (1.01325 bar, 300 K) ensemble by a Nosé–Hoover Langevin barostat (46, 47) and a Langevin thermostat (48) with a damping coefficient 1 ps^−1^. The final trajectories were used to analyze residue pair energy analysis.

### Residue pair energy calculation

Pairwise residue energy analysis was performed by gRINN (49), for representative frames of each cluster after *Cα* principal component analysis of MD trajectories and clustering on the 2D projections using the mean shift algorithm.

### Statistical analysis

Statistical analysis was performed using custom python scripts and the scipy.stats package. All the code to process data and generate figures will be publicly available.

## Results

### Peptide substitution scans for naturally elicited FP-directed HIV-1 Env-neutralizing antibodies reveal improved binding to non-canonical FP sequences

**Figure 1:**
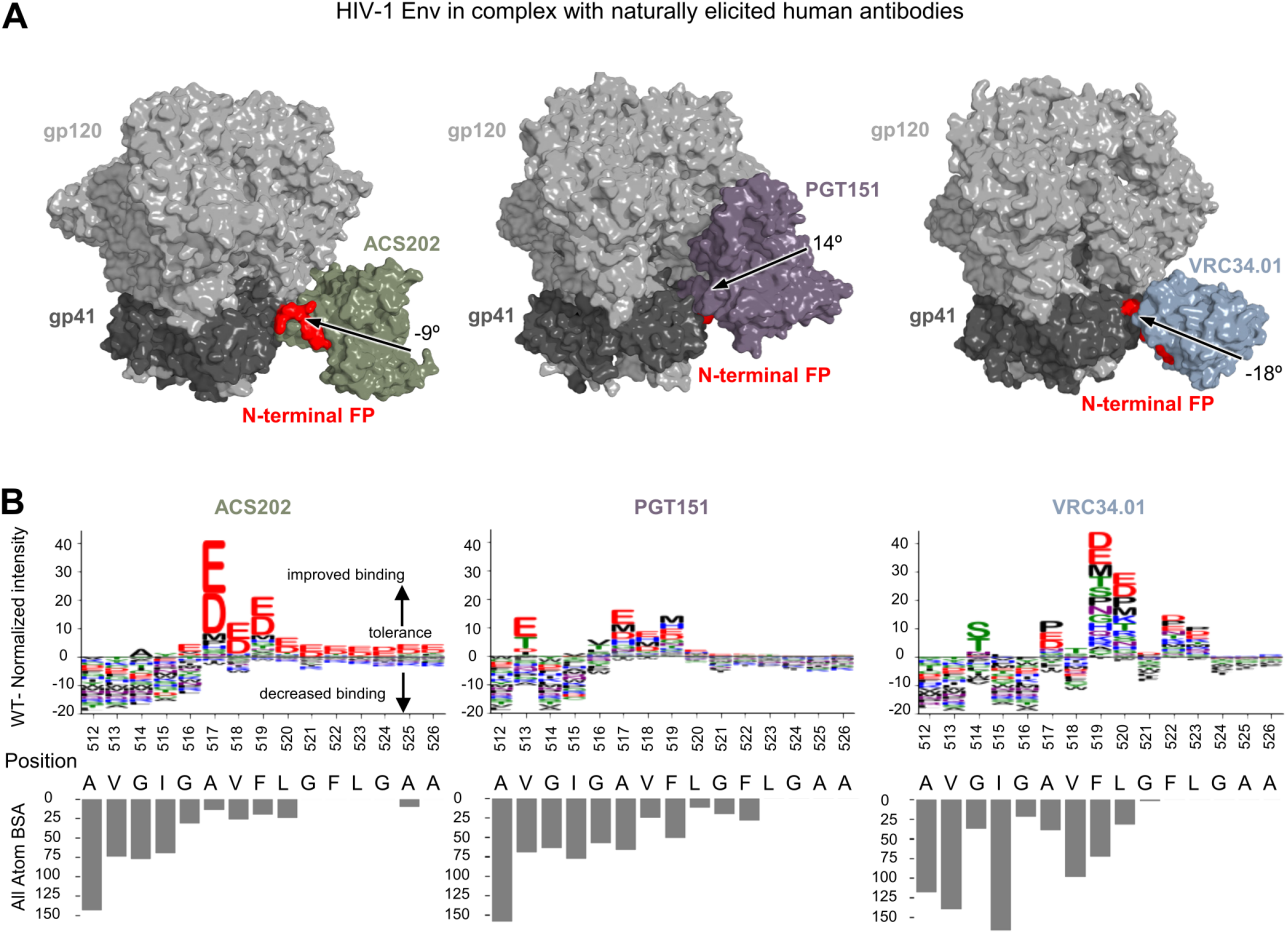
Diverse FP sequences are target sites for naturally elicited antibodies, as revealed by peptide-substitution analysis. **(A)** Structural reprentation of the HIV-1 Env trimer in complex with naturally elicited FP-targeted antibodies.The angle of approach was calculated using the trimer axis ,and the antibody axis. The antibody axis was calculated by measuring the rotation of the light chain onto the heavy chain during a superposition alignment. **(B)** Logo plots with peptide substitution scan data from PEPperPRINT with all atom BSA bar plots.The height of each amino acid mutation corresponds to the relative improvement over WT, with positive values improving over WT, negative values decreasing binding over WT.

Naturally elicited, FP-directed, broadly neutralizing antibodies, VRC34.01, PGT151, and ACS202, assume multiple angles of approach when binding the HIV-1 trimer at the FP site (**Figure 1A**). To provide insight into sequence requirements for binding, epitope substitution scans were performed with PEPperPRINT FP15 peptide microarrays (**Figure 1B**). These scans revealed that all three naturally elicited human antibodies could bind diverse FP sequences.

ACS202 tolerated single substitutions at multiple positions (514–526). ACS202 analysis displayed a conserved N-terminal core A_512_VGIG_516_, a highly variable A_517_VF_519_ region with a preference for the negatively charged residues Asp and Glu with a 10-fold binding intensity increase with respect to the WT, as well as the hydrophobic residues Pro and Met to a much lower degree. The C-terminal region L_520_GFLGAA_526_ tolerated most mutations and displayed a consistent preference for negatively charged residues over WT. Overall conserved regions correlated well with the all-atom buried surface area (BSA).

PGT151 on the other hand showed a more moderate increase in normalized intensities at a few selected positions 513 and 516–520. The N-terminal core A_512_VGIG_516_ remained conserved except for position 513 where Glu, Thr, Asp, and Ile were preferred over WT. The central region was once again highly variable with position 516 preferring Val and Thr. Most substitutions in the A_517_VF_519_ region improved over the WT. The C-terminal region L_520_GFLGAA_526_ tolerated most mutations but did not show the strong improvement with negatively charged residues as shown in ACS202.

The third naturally elicited antibody N123-VRC34.01 (referred to as VRC34.01) showed strong conservation and preference for WT residues at positions 512–513 and 515–516. Mutations at position 514 were slightly more tolerated, with a strong preference for Ser and Thr residues and a lower BSA than its neighbors. Similarly, mutations in position 517 allowed for certain flexibility with strong improvement over WT with Pro, Glu, and Asp mutations. Positions 519 and 520 not only tolerated all single amino-acid substitutions but also improved over WT.

Overall, naturally elicited antibodies displayed a conserved A_512_VGIG_516_ region with some exceptions while tolerating most substitutions in the C-terminal region. Multiple non-canonical mutants significantly improved binding over WT, with a strong preference for charged residues which are not usually found in actual HIV sequences.

### Peptide substitution scans for vaccine-elicited FP-directed HIV-1 Env-neutralizing antibodies reveal lower tolerance to FP variability in the N-terminal region

**Figure 2:**
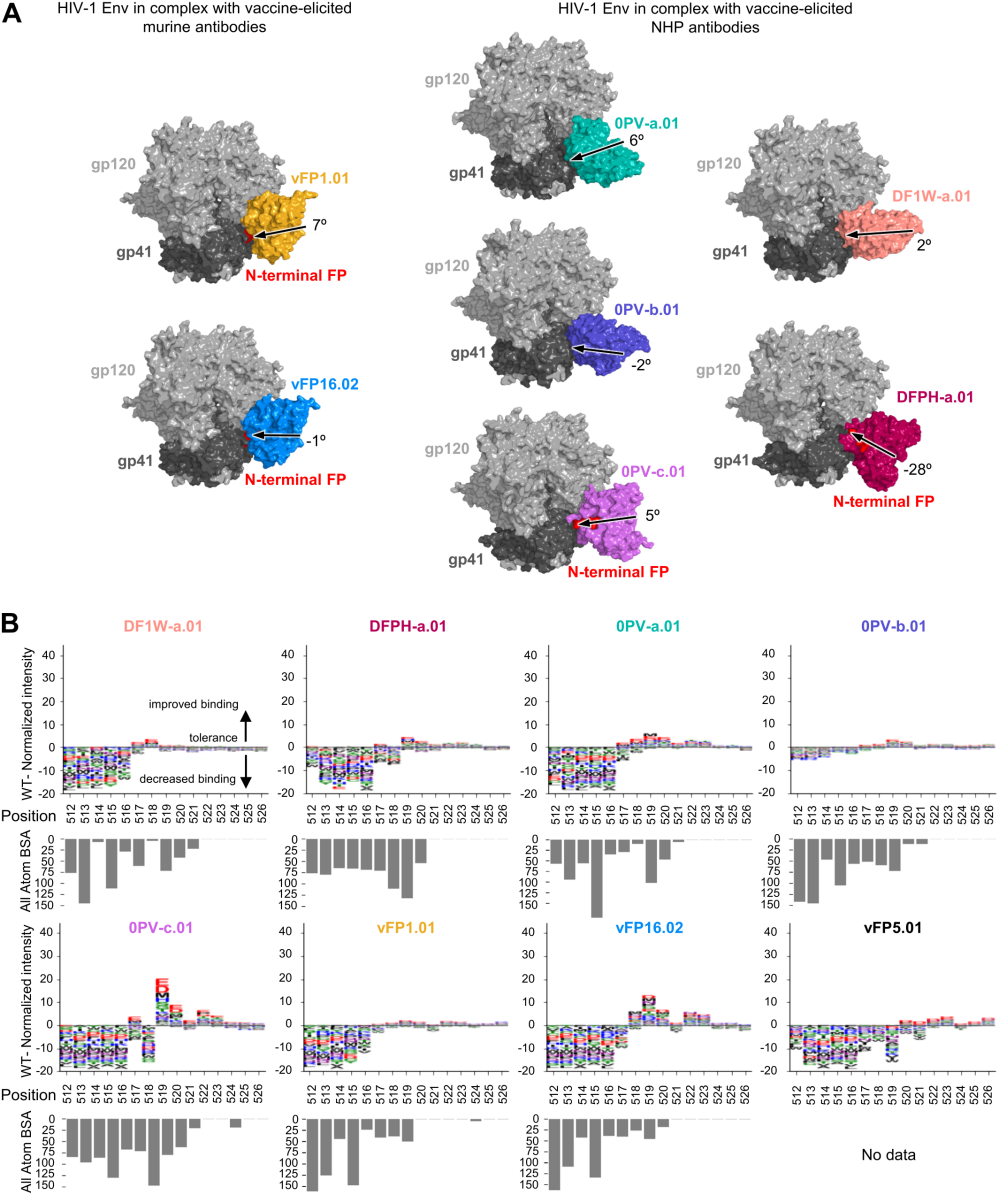
Peptide-substitution analysis reveals vaccine-elicted antibodies targeting FP site of vulnerability are less permissive to FP variation **(A)** Overall structure context of vaccine elicited antibodies in complex with HIV-1 Env trimer. The structure from DFPH-a. 15 is displayed for DFPH-a.01. The angle of approach was calculated using the trimer axis, and the antibody axis. The antibody axis was calculated by measuring the rotation of the light chain onto the heavy chain during a superposition alignmnent. **(B)** Logo plots with peptide substitution scan data from PEPperPRINT with all atom BSA bar plots. The heightof each amino acid mutation corresponds to the relative improvement over WT, with positive values improving over WT, negative values decreasing binding over WT. DFPH-a.01 BSA values were calculated using the closely related DFPH-a.15 structure as the DFPH-a.01 structure was unavailable.

While vaccine-elicited FP-directed murine NAbs vFP16.02 and vFP1.01 assume similar angles of approach when binding the HIV-1 trimer at the FP site (**Figure 2A**), neutralizing antibodies elicited in NHP assume multiple angles of approach on the trimer (**Figure 1A**). Epitope substitution scans were performed with PEPperPRINT FP15 peptide microarrays (**Figure 2B**). Overall, these scans revealed a lower tolerance than naturally elicited antibodies for single mutations in the N-terminal core A_512_VGIG_516_, suggesting that WT residues remain key for binding. The only exception was 0PV-b.01, which showed reduced binding responses slightly above the noise level of the assay. There was clear promiscuity in the A_517_VFLGFLGAA_526_ region without significantly improved binders over WT for DF1W-a.01, DFPH-a.01, 0PV-b.01, and vFP1.01. The V_518_FL_520_ region showed moderate improvement over WT for 0PV-a.01 and vFP16.02. Interestingly 0PV-c.01 showed great improvement at positions 519 and 520 but did not tolerate mutations at position 518.

Overall, the vaccine-elicited antibodies appeared to be more optimized for the most common FP sequence in the N-terminal region (residues 512 through 516). In the 517–520 region, residue alterations did not substantially affect binding. Some of the similar substitutions that substantially improved affinity for naturally elicited antibodies also improved affinity for the vaccine-elicited antibodies, but to a much lower degree. In addition, the C-terminal region (521–526) generally showed little interaction with antibodies, and most substitutions had little impact.

### Statistically significant difference in diversity of FP sequences bound by vaccine elicited and naturally elicited antibodies

**Figure 3:**
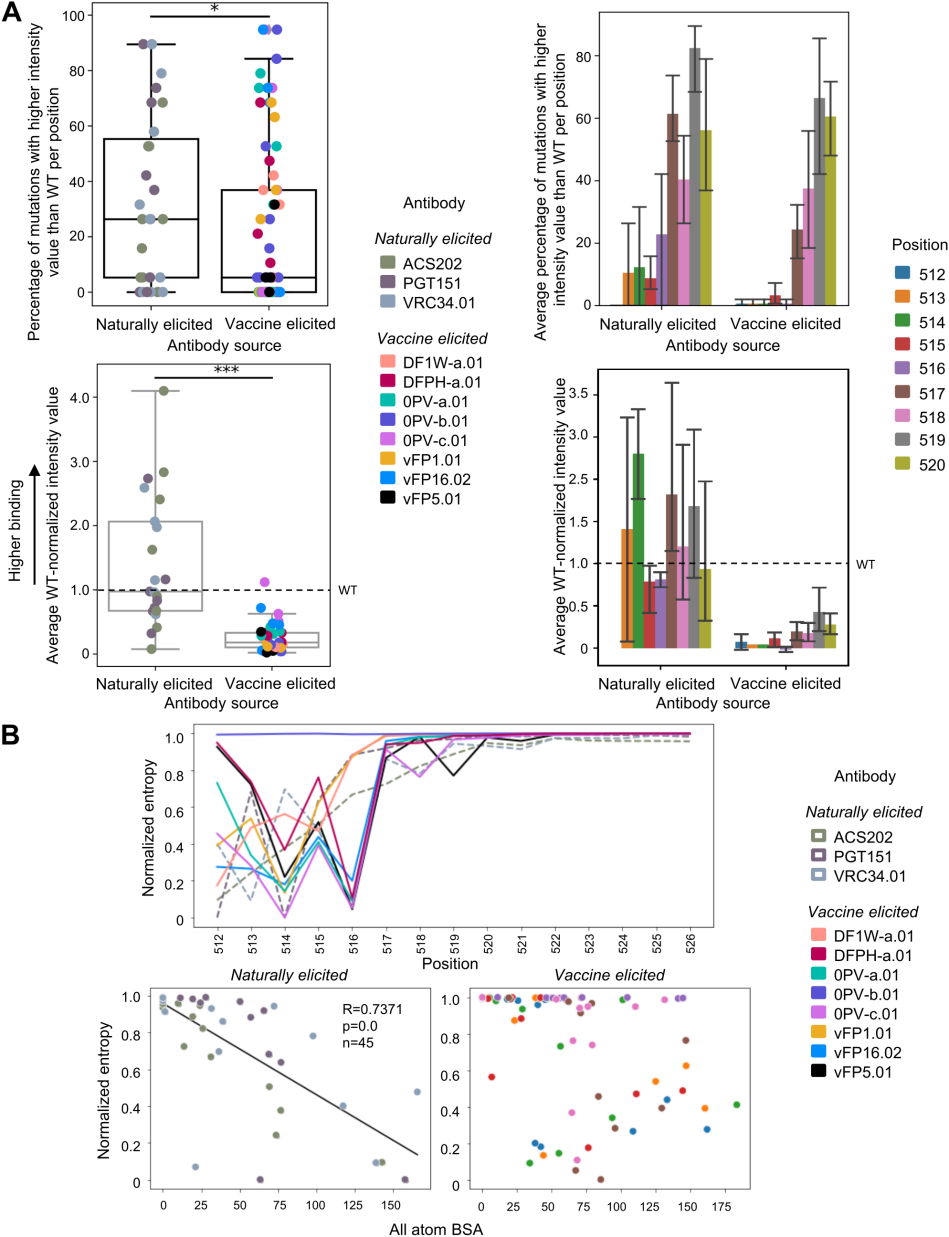
Quantification of FP recognation reveals naturally elicited antibodies bind significantly more diverse FP sequences than those that are vaccine-elicited **(A)** Top oanels: Count analysis: Number of mutation with higher PEPperPRINT intensity value than WT for positions 512-520. On the left, each point corresponds to a particular position and antibody for n=72 vaccine elicited and n=27 for naturally elicited antibodies. On the right: values are averaged across antibodies for a given position. Bottom panels: Magnitude analysis: Average PEPperPRINT intensity value for entries higher than WT for positions 512-520. Error bars correspond to 95% confidence interval. **(B)** The intensity values for each position are normalized and used to compute per-position entropies with base=20 to normalize. Entropies for both naturally and vaccine elicited antibodies are correlated with all atom BSA.

To quantitively compare naturally and vaccine-elicited antibodies, the number of PEPperPRINT binding intensity values for the 19 mutants *I_mut_* higher than the WT value *I_WT_* were counted after grouping by FP position and antibody (**Figure 3A** top panel). Position 512 was absolutely conserved for both vaccine and naturally elicited antibodies. For positions 513–516, almost no mutations improved binding to vaccine-elicited antibodies whereas 10%–20% of mutants improved binding to naturally elicited antibodies. Mutations in the 517–520 region display very high levels of improvement (around 50%) for both types of antibodies.

The magnitude of the responses *M* was also assessed by averaging the WT-normalized PEPperPRINT binding intensity values *I* per FP position *i* across all 19 amino acid substitutions, as shown by $M=\frac{1}{20}\sum_{i}\frac{I_{mut_{i}}-I_{WT_{i}}}{I_{WT_{i}}}$ (**Figure 3A** bottom panel). Here, significant differences were observed between naturally and vaccine-elicited antibodies for all positions except for position 512 which was highly conserved, as well as position 520.

The distribution of PEPperPRINT intensities was used to compute the entropy for a given position (**Figure 3B**). Positions 518 to 526 displayed the maximum entropy for both vaccine and naturally elicited antibodies, suggesting a uniform distribution over all possible mutations. Naturally elicited antibodies displayed high correlation between entropy and all-atom BSA, with low entropy positions having the highest BSA values (R=0.73, P<1e−5). In other words, exposed residue positions showed little preference to mutations whereas buried positions had specific preferences for certain mutations. However, some clear exceptions exist; positions such as 517 in complex with ACS202 can be easily mutated while having very low BSA of 10 A^2^, whereas 519 in VRC34 has a high BSA of 75 A^2^ and tolerated most substitutions. Surprisingly, vaccine-elicited antibodies did not seem to follow such a trend, with very different clusters for different positions. The strong preference to certain residues regardless of the antibody contacts might relate to the initial priming during vaccination.

### PEPperPRINT binding data for FP antibodies correlates significantly with other established metrics including differential selection and binding in trimer context by Octet

To check if the effects of FP mutations on antibody binding observed above with the PEPperPRINT data would remain true in the trimer context, we created BG505 trimers with selected mutations and analyzed their binding to antibodies by Octet (**Supplementary Figure S1A**). In particular, mutation A517E was selected since it strongly improves binding across all naturally elicited antibodies while maintaining a small change in vaccine-elicted antibodies. Mutation G514S was also selected since it strongly and uniquely improves binding with the VRC34.01 antibody. The binding response to each antibody was measured in triplicates for the mutant trimers compared with the BG505 trimer with the WT FP sequence (BG505_RnS). The mutation A517E improved binding to all antibodies, except for VRC34.01 and DF1W-a.01. The G514S mutation improved VRC34.01 binding, consistent with that observed in the PEPperPRINT data, but it also increased binding to DF1W-a.01, contradicting PEPperPRINT prediction in the peptide context. Overall, the effects of FP mutations measured by PEPperPRINT in the context of peptide showed a medium correlation (R=0.6828, p=0.00179) with those measured by Octet in the trimer context (**Supplementary Figure S1B**).

In addition to the correlation with Octet binding in the trimer context, PEPperPRINT binding values correlated with differential selection metrics (24, 25) (**Supplementary Figure S2**). Because differential selection metrics maps the viral escape mutations from bnAb neutralization, this correlation indicated that PEPperPRINT binding values correlated with neutralization tolerance of the mutations. The effect of individual mutations was also consistent between the PEPperPRINT intensity and the corresponding differential selection value. For example, for PGT151, almost all amino-acid substitutions at residues 512 and 514 had negative PEPperPRINT intensities (**Figure 1B**), and these two positions have the most escape mutations (25); position 513 have relatively less differential selection, and Glu, Thr, and Asp are not among the escape mutations at this position. For VRC34.01, positions 512–516 have high differential selection, with 514 slightly lower without Ser in the escape mutations (50), mostly consistent with the PEPperPRINT intensities in the FP region. Vaccine-elicited antibodies, DF1W-a.01, 0PV-a.01, 0PV-b.01, DFPH-a.01, and vFP16.02, also showed PEPperPrint intensities for FP sequence screening that were consistent with the differential selection data, with positions 512–516 having many escape mutations (18, 50).

### Cryo-EM structure of ACS202 in complex with the BG505 Env trimer with A517E explains enhanced affinity of A517E substitution

**Figure 4:**
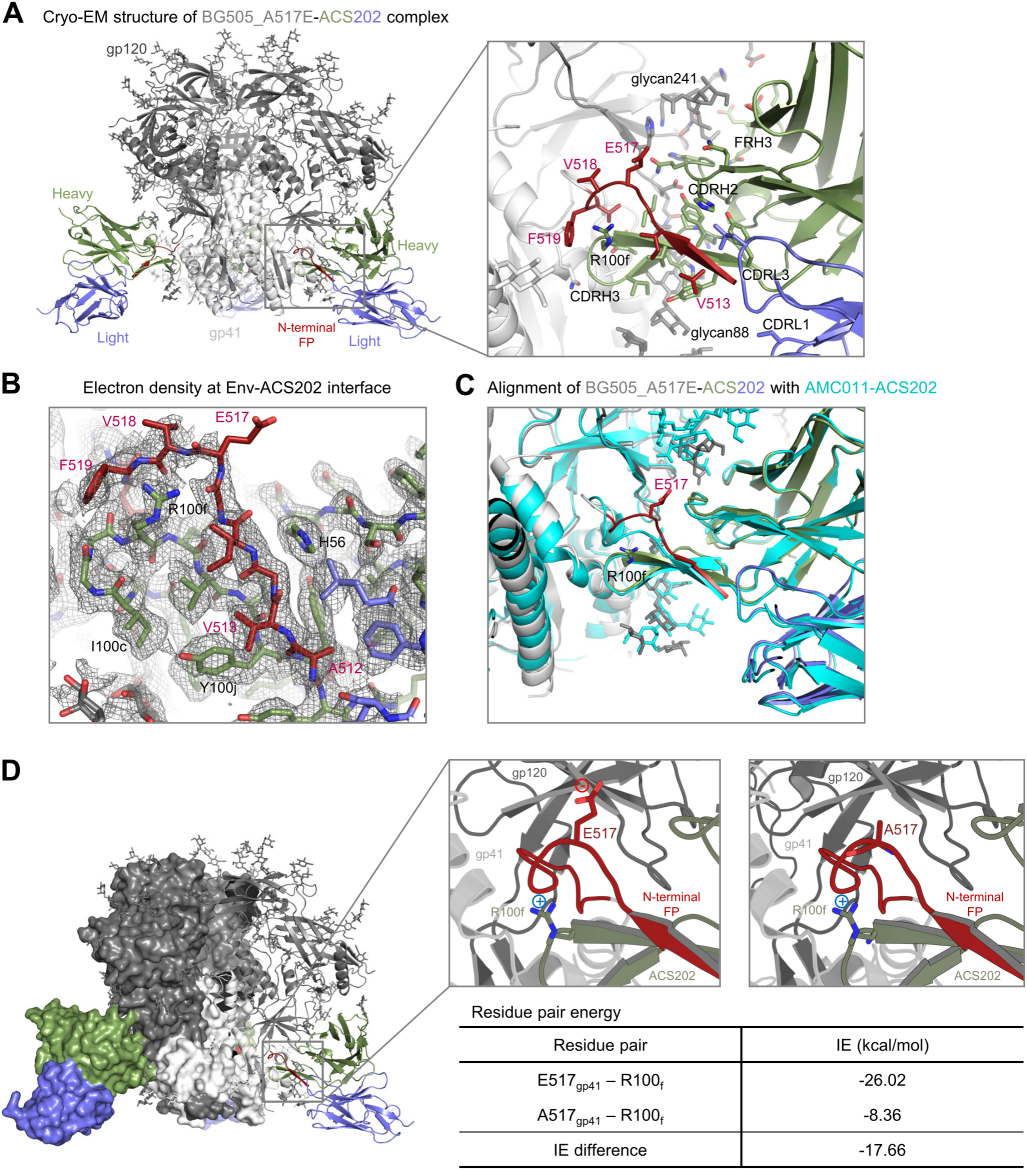
Cryo-EM structure of ACS202 in complex with BG505 Env trimer with A517E explains enhanced affinity of A517E substitution **(A)** Cryo-EM structure of BG505_A517E in complex with ACS202. **(B)** Electron density at the trimer-antibody interface,contoured at 4 *σ* The map was from final cryoSPARC non-uniformed refinement,sharpened, at a nominal resolution of 2.3 Å. **(C)** Structural alignment of BG505_A517E-ACS202 complexes. The structures were aligned by the antibody heavy chain shown. **(D)** Key residue pair in the BG505_ACS202 complex contributed to enhanced binding affinity.

To elucidate the structural mechanism for the improvement of antibody binding to the A517E mutant, we determined a cryo-EM structure of ACS202 Fab in complex with BG505_RnS_7mut_A517E, a prefusion-stabilized Env trimer (51) with the alanine at position 517 mutated to glutamate. We obtained a 3D-reconstruction map at 2.30 Å from 635,332 particles applying C3 symmetry (**Supplementary Figure S3**; **Supplementary Table S1**). Local resolution estimation indicated that the antibody bound with substantial flexibility, with Fab having a substantially lower resolution than the Env trimer (**Supplementary Figure S3E**). However, the antibody–antigen interface was well defined in the density map and showed that ACS202 interacted with the N-terminal segment of FP through β-strand interactions from its third complementarity determining region of heavy chain (CDR H3) (**Figure 4A**), similar to that observed for the previously determined ACS202–AMC011 complex (32). The electron density was relatively weak around residue 517, especially around the glutamate side chain (**Figure 4B**), indicating conformational flexibility. Structural alignment with the ACS202–AMC011 complex (PDB: 6nc2) (32) indicated that A517E mutation likely induced local conformational flexibility. AMC011 has the N-terminal 15 residues of FP identical to that in BG505, with alanine at residue 517. The two structures were aligned by one heavy chain variable domain of ACS202, with an RMSD of 0.677 Å for 126 aligned Cα atoms (**Figure 4C**). Both heavy and light chains, as well as most of the protomers (gp41 and gp120) interacting with the aligned antibody, were well aligned. However, residues 517–518 had substantial shifts, with main-chain atoms between the two structures having up to ~4 Å apart. The conformational flexibility around E517 could potentially allow the nearby R100f side chain on CDR H3 to have more favorable interactions with the carbonyl groups of residues 516–517 or to have dynamic charge–charge interactions with the E517 side chain, although the refined cryo-EM structure exhibited a distance of 7.8 Å between the two oppositely charged side chains. Structural constraints from the rest of the trimer–antibody interactions likely limit the interactions between the two side chains. This is consistent with our observation that PEPperPRINT analysis of amino acid substitutions in the FP, which was not constrained by trimer interactions, exhibited substantial enhanced binding to ACS202 with glutamate or aspartate at residue 517 (**Figure 1B**), whereas when measured by Octet in the context of trimer, the A517E mutation exhibited a much smaller increase in binding to ACS202 (**Supplementary Figure S1**).

To confirm the dynamic interactions between the side chains of E517 at FP and R100f of CDR H3, we conducted an analysis of the energetic interactions between these residue pairs using the trajectory obtained from molecular dynamics simulations (**Figure 4D**). Pairwise residue energy was calculated between R100f and residue 517 as either alanine or glutamate, and a decrease of 17.66 kcal/mol was observed for the A517E mutation, indicating that there were favorable dynamic interactions between residues R100f and E517.

Overall, the cryo-EM structure of the BG505_A517E in complex with ACS202 revealed that, in the context of trimer–antibody binding interactions, FP was unable to adopt a stable conformation that brought the side chains of E517 and R100f close enough for salt-bridge interaction. However, the A517E mutation induced conformational flexibility around residue 517 in FP of the trimer–antibody complex, allowing for a favorable interaction between residues E517 and R100f, contributing to the observed improvement in affinity, although in a smaller scale than that observed for the free FP binding to the antibody ACS202. This favorable dynamic interaction could be between the two side chains or between the R100f side chain and the main-chain carbonyl groups of residues 517–518.

### Atomic-level interactions from MD simulation analysis corroborate trends observed with PEPperPRINT intensity values

**Figure 5:**
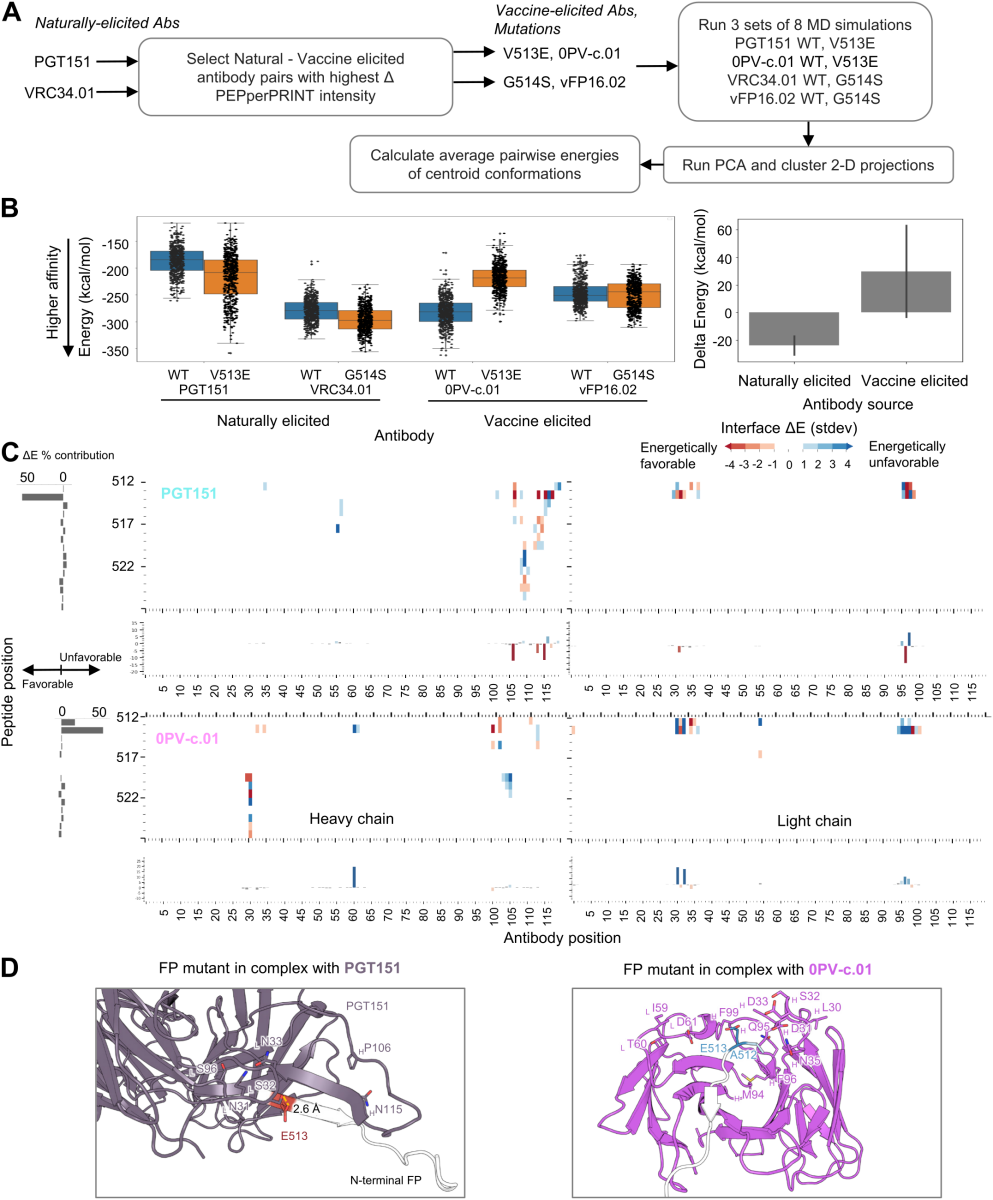
Atomic level interactions from MD simulation analysis corroborate trends observed by peptide-substitution analysis **(A)** Diagram for *in silico* structural analysis pipeline of FP mutants. **(B)** Overall energies suggest mutations on naturally elicited antibodies improve binding whereas vaccine elicited antibodies do not tolerate those mutations as well **(C)** Pairwise energy analysis reveals specific interactions responsible for the binding differences observed in PEPperPRINT Numbering of residues is sequential and not in kabat format **(D)** MD structures of FP mutants in complex with antibodies FP residues are color coded by the energy contributions from **(C)**, with red indicating more favorable energetic interactions and blue indicating unfavorable.

To further understand the structural mechanisms explaining the improved binding to naturally elicited antibodies, we selected two mutations and analyzed the trajectories resulting from MD simulations. For the mutant selection, we first calculated the delta PEPperPRINT intensities Δ*I* independently for the vaccine-elicited antibodies VRC34.01 and PGT151 by subtracting the average naturally elicited intensity values for each position and mutation (**Figure 5A**; **Supplementary Figure S4A**). The highest Δ*I* values corresponding to the highest expected differences between vaccine and naturally elicited antibodies were G514S for VRC34.01 and V513E for PGT151. WT and mutated versions of the FP were generated in complex with the corresponding naturally elicited antibodies and the vaccine-elicited antibodies 0PVc.01 and vFP16.02, which also showed the highest Δ*I* values (eight total complex structures). The structures were used to run three independent sets of MD simulations. The resulting trajectories were analyzed by running PCA on C-alpha atoms and clustered on the 2D projections using the mean-shift algorithm (52). The centroid of each cluster was computed, and the closest MD frame to the centroid by Euclidean distance was used as a representative frame (**Supplementary Figure S4B**). These frames were then used to compute the average pairwise energies of centroid conformations. The average pairwise energies were used to compare between WT and mutated forms by subtracting the pairwise energies resulting in Δ*E* maps (**Figure 5C**; **Supplementary Figure S5**).

From the PEPperPRINT Δ*I* values, we expected an increased binding for naturally elicited antibodies and a decrease in mutated residues for vaccine-elicited antibodies. The interaction energy between the antibody and FP complex was computed using NAMD. The expected trend is observed for the naturally elicited antibodies PGT151 and VRC34.01, where the V513E and G514S mutants show a slight decrease in energy from an average of −186 kcal/mol to −217 kcal/mol and from −279 kcal/mol to −296 kcal/mol, respectively (**Figure 5B**). For PGT151, a V513E mutation introduced a strong hydrogen bond interaction with a serine residue in CDRL1 (**Figure 5D**). The most dramatic and consistent change across all antibodies was observed for 0PVc.01, with V513E mutation increasing the energy from −282 kcal/mol to −219 kcal/mol. Three aspartic acid residues within the paratope were identified as energetically unfavorable. The vFP16.02 showed a negligible decrease with mutation G514S from −246 kcal/mol to −250 kcal/mol. Overall, the naturally elicited antibodies show the expected trends of lowering the interaction energies although with significant overlap across the full trajectories.

## Discussion

The vaccine elicitation of antibodies capable of broad and potent neutralization of HIV-1 is still at a nascent stage. Antibodies isolated from lineage-based vaccine strategies have achieved substantial breadth in knock-in mice (8, 9), whereas antibodies from epitope-based strategies have achieved over 50% breadth only against FP site of vulnerability and only with low serum titers (18). Because antibodies elicited by the latter strategy derived from different antibody classes than the template naturally elicited antibodies, we felt it would be important to assess the similarity by which naturally elicited and vaccine-elicited antibodies recognized FPs.

Vaccine-elicited antibodies showed preference for the most common FP sequence, with little tolerance to FP sequence variations, whereas naturally elicited antibodies showed more tolerance to FP sequence variations, with some FP mutations strongly improving antibody binding. This is likely to be due to vaccine-elicited antibodies being induced by immunogens that contain only the most common FP sequence, whereas in natural infection, the immune system is exposed to HIV-1 viruses that constantly mutate, likely with various FP sequences. In addition, we note that vaccine-elicited antibodies tend to be closer to germline sequences than naturally elicited antibodies (15, 18, 21, 23), which could explain a decrease in mutation tolerance. However, other factors might influence the observed differences. Vaccine-elicited antibodies are derived from immunizations with the engineered, stabilized ectodomain of Env trimers, whereas naturally elicited antibodies are induced with Env trimers that are membrane bound on the HIV-1 virions. Vaccination with native-like Env trimers as recently described (53–55) could be important to restore the observed flexibility of naturally elicited antibodies in recognizing various FP sequences, as native-like Env trimers could potentially present FP epitope similarly to viral infection.

In addition, the trajectories from MD simulations for selected FP mutations suggest lower interaction energies with naturally elicited antibodies, and higher interaction energies to vaccine-elicited antibodies, consistent with the observed binding values from the substitution scan analysis. Although statistically significant differences were found between naturally and vaccine-elicited antibodies, it is still possible that the observed differences are caused by the different origins of these antibodies (human versus mice/macaque). The number of characterized naturally elicited (3) and vaccine-elicited (8) antibodies was also less than optimal, limited by the few naturally elicited antibodies against FP that have thus far been identified.

It will be interesting to see if the revealed differences in FP recognition can be used to improve the breath and potency of vaccine-elicited antibodies. In this context, we note that differences in FP recognition have been used to improve the neutralization breadth of antibody VRC34.01 from 51% to 79% (56) and that it was interesting to observe that, while naturally elicited antibodies often have superior potency, they were not fully optimized in terms of binding to the most commonly occurring FP15 sequence. The fact that similar changes at residues 517–520 positively impacted the recognition of naturally elicited antibodies (and to a lesser degree the vaccine-elicited ones) suggests that antibodies have not fully optimized their electrostatics interactions. The much lower enhancement in affinity noted with the vaccine-elicited antibodies does suggest, however, that they were optimized to recognize exposed N-terminus of FP, perhaps a consequence of their priming with the most common FP sequence, as opposed to the variable FP sequences found in natural HIV-1 infection for naturally elicited antibodies.

## Data Availability

The datasets presented in this study can be found in online repositories. The names of the repository/repositories and accession number(s) can be found below: https://doi.org/10.2210/pdb8TOX/pdb; https://www.emdataresource.org/EMD-41461.
