# Supplementary material for: Vaccine-elicited and naturally elicited antibodies differ in their recognition of the HIV-1 fusion peptide
Source: Front Immunol. 2024 Nov 14;15:1484029. doi: 10.3389/fimmu.2024.1484029 (PMC11602501; doi:10.3389/fimmu.2024.1484029)
Supplement: Supplementary file 1 [file DataSheet1.pdf]

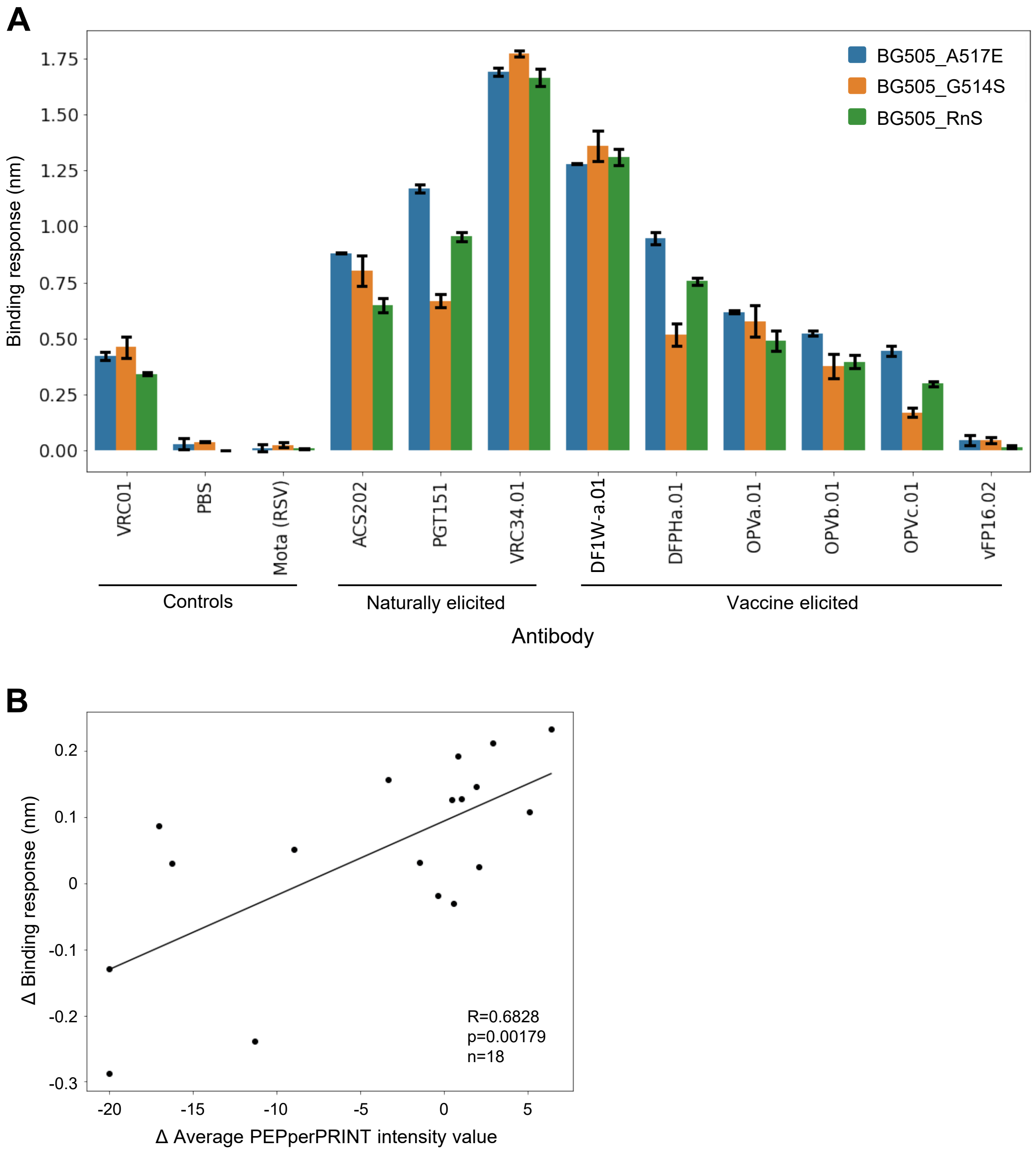

Figure S1. Octet binding in trimer context correlates with PEPperPRINT binding in peptide context

(A) Octet binding data for selected antibodies for WT and two mutations (G514S, A517E) with high PEPperPRINT intensity values.

(B) Correlation between octet binding data in trimer context and PEPperPRINT intensity values normalized by mean value per position.

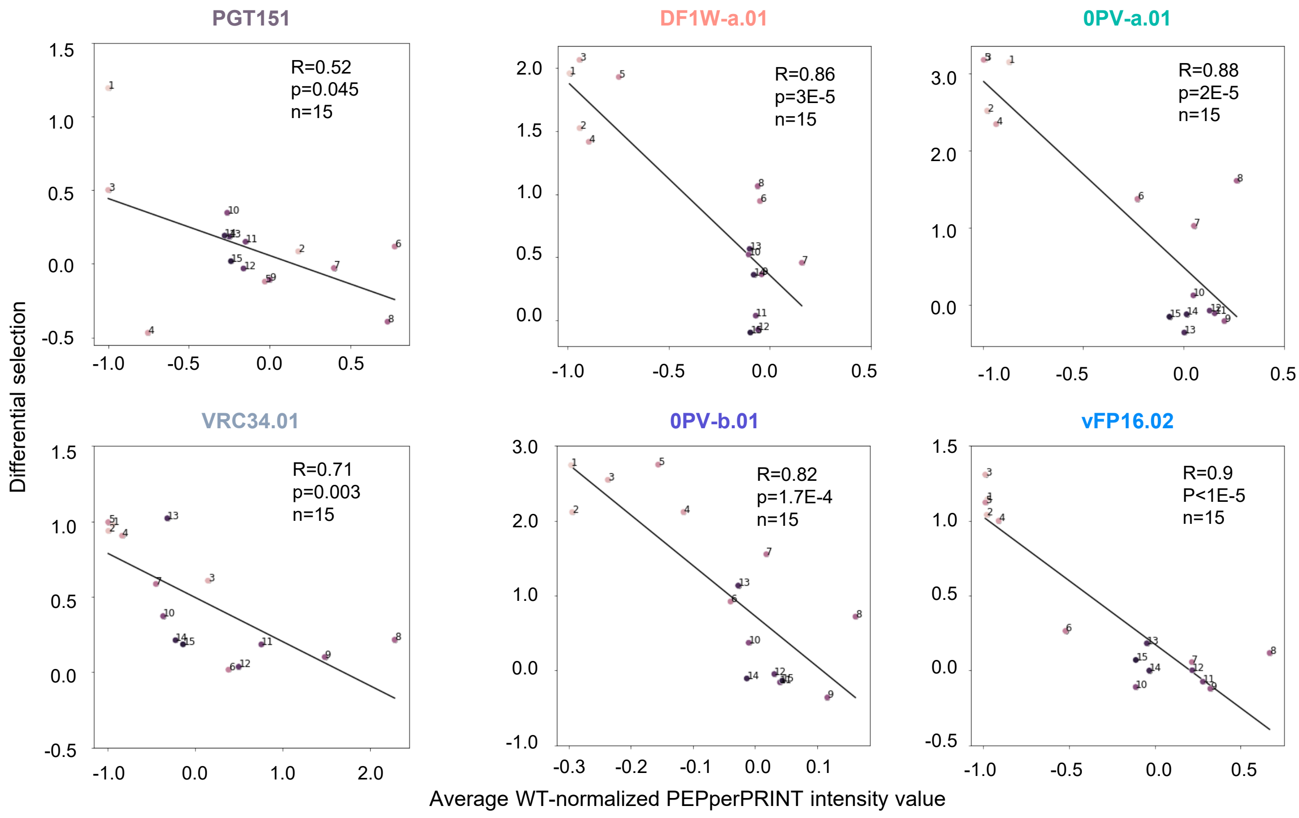

Figure S2. Peptide-substitution analysis correlates with differential selection measures from deep mutational scanning
PEPperPRINT WT-normalized intensity values correlate with differential selection values, averaged across site for naturally and vaccine elicited antibodies. Numbers correspond to FP position with 1 corresponding to position 512.

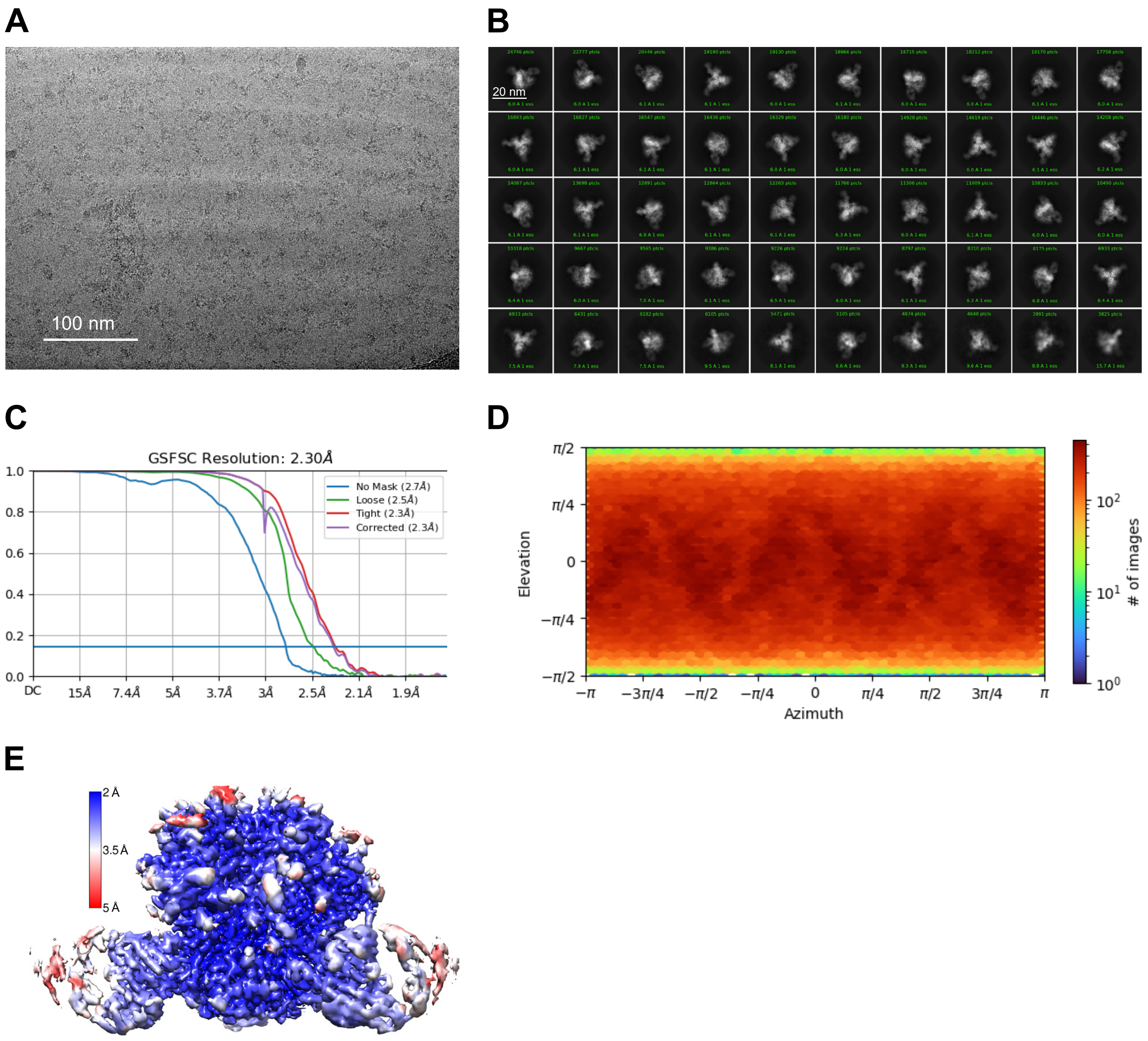

Figure S3. Cryo-EM validation for ACS202 in complex with BG505_RnS_7mut_A517E

(A) Representative micrograph.

(B) Selected 2D classes.

(C) Fourier shell correlation (FSC) curves showing gold-standard FSC resolution at 2.30 Å.

(D) Orientations of all particles used in the final refinement shown as a heatmap.

(E) 3D reconstruction density map at 2.3 Å from non-uniform refinement with C3 symmetry, with local resolution indicated by color gradient.

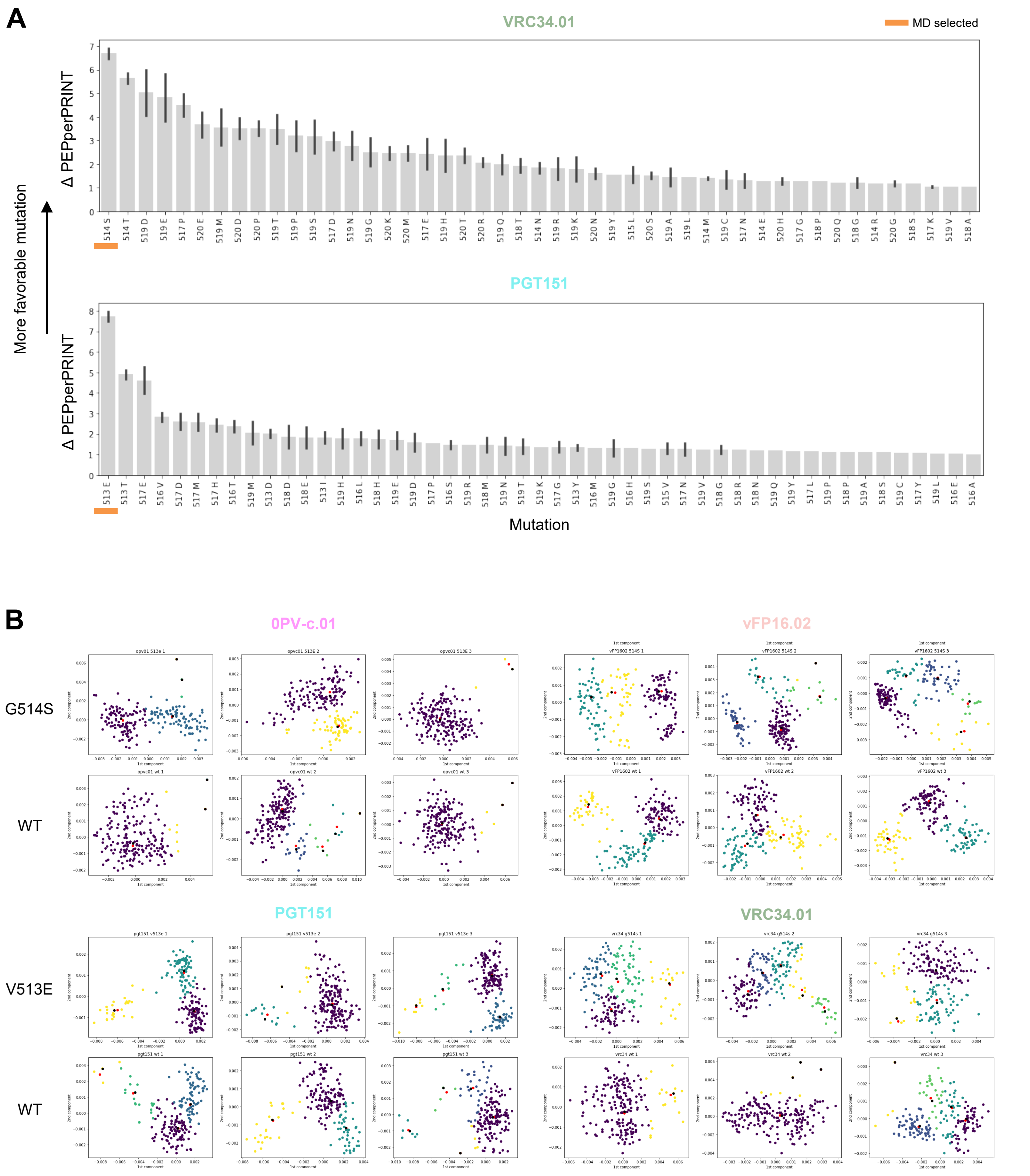

Figure S4. MD analysis pre-processing

(A) Mutant selection for MD simulations by computing the highest ΔPEPperPRINT value between a given naturally elicited antibody value and the mean vaccine elicited antibody value.

(B) PCA analysis of the MD simulations frames from C-α and clustering with the mean shift algorithm. The cluster centroid is in red, and the closest frame to the centroid selected for pairwise analysis in black.

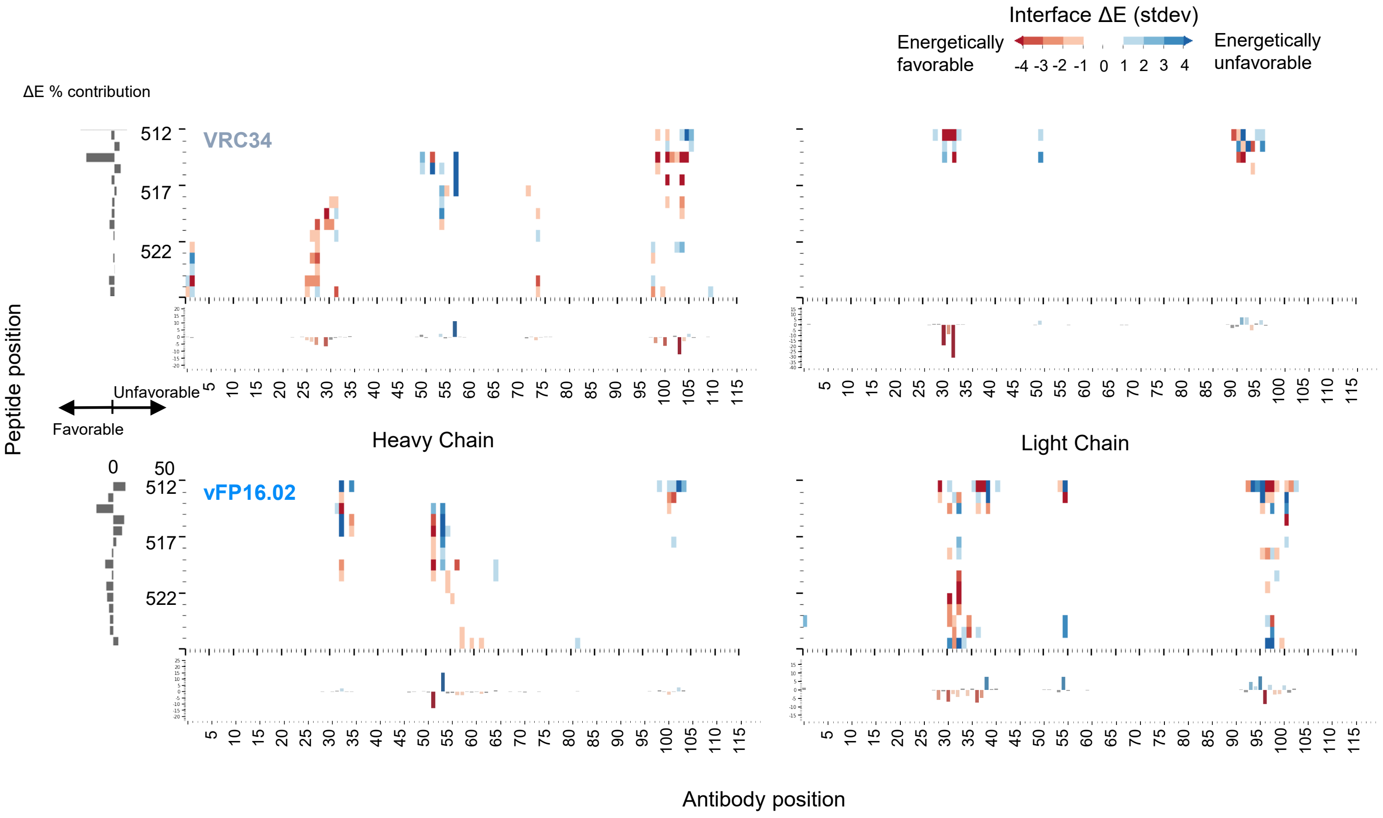

Figure S5. Atomic level interactions from MD simulation analysis corroborate trends observed by peptide-substitution analysis

Pairwise energy analysis reveals specific interactions responsible for the binding differences observed in PEPperPRINT. Numbering of residues is sequential and not in Kabat format.

Table S1. Cryo-EM data and refinement statistics for BG505_RnS_7mut_A517E in complex with ACS202

**EMDB ID**	EMD-41461
**PDB ID**	8TOX
Data collection	
Microscope	FEI Titan Krios
Voltage (kV)	300
Electron dose ($e^-/Å^2$)	54.40
Detector	Gatan K3
Pixel size (Å)	0.415/0.83
Nominal defocus range (μm)	-0.7 to -2.0
Magnification	105,000
Reconstruction	
Software	cryoSPARC v3.3.1
Particles	635,332
Symmetry	C3
Resolution (Å) ($FSC_{0.143}$)	2.3
Refinement	
Software	Phenix 1.20
Protein residues	2490
Water	369
Ligands	BMA: 18; NAG: 114; MAN: 27
CC (box)	0.82
CC (mask)	0.85
R.m.s. deviations	
Bond lengths (Å)	0.003
Bond angles (°)	0.503
Validation	
Molprobity score	1.31
Clash score	3.52
Rotamer outliers (%)	0.51
Ramachandran	
Favored regions (%)	97.06
Allowed regions (%)	2.94
Disallowed regions (%)	0
